# Malakoplakia of the appendix, an uncommon entity at an unusual site: a case report

**DOI:** 10.1186/1752-1947-2-181

**Published:** 2008-05-29

**Authors:** Sameer S Shaktawat, Mark CJ Sissons

**Affiliations:** 1 Department of Histopathology, Central Manchester and Manchester Children's University Hospital NHS Trust, Oxford Road, Manchester M13 9WL, UK; 2 Blackpool Victoria Hospital, Whinney Heys Road, Blackpool, Lancashire FY3 8NR, UK

## Abstract

**Introduction:**

Malakoplakia is an uncommon inflammatory condition usually affecting the genitourinary tract, which has been associated with infections, tumours and immunocompromised states.

**Case presentation:**

We report a case of malakoplakia in the appendix of a 61-year-old man with a long-standing history of ulcerative colitis. Clinically and macroscopically malakoplakia can simulate tumours or abscesses and can cause diagnostic difficulties. Histologically malakoplakia in the gastrointestinal tract must be differentiated from Whipple disease, other infectious and noninfectious granulomatous disorders and histiocyte storage diseases. To the best of our knowledge, this is the first case of malakoplakia of the appendix reported in association with ulcerative colitis and the sixth reported case of malakoplakia of the appendix in the literature. Although the underlying disease in our case was ulcerative colitis, the malakoplakia was limited to the appendix.

**Conclusion:**

The significance of this finding is not clear but we feel that this was a localised manifestation of the underlying immunosuppressive state. Ulcerative colitis and treatment with steroids may make a patient immunosuppressive and the local and systemic change in the immunity may facilitate the proliferation of the organisms and modify the phagocytic abilities of the macrophages.

## Introduction

Malakoplakia is an inflammatory reaction to organisms, which include bacteria, mycobacteria, fungi and occasionally parasites. Malakoplakia can simulate tumours and may result in diagnostic difficulties. Usually occurring in the urinary tract, it has been described in almost all organs. We describe a case of malakoplakia of the appendix, which is the first case described in association with inflammatory bowel disease and overall the sixth case reported in the medical literature.

## Case presentation

A 61-year-old male with a 20-year history of ulcerative colitis presented with increasing abdominal pain, diarrhoea and rectal bleeding resulting in a proctocolectomy with end-ileostomy.

The resected total colectomy specimen consisted of 90 cm of colon, 5 cm of terminal ileum with a 6 cm appendix. The entire colonic mucosal surface was haemorrhagic with pseudopolyps. There was focal massive dilatation of the colon and black-green discolouration. The appendix was enlarged with adherent omentum, measuring 6 × 3 × 3 cm^3^. The cut surface showed several tiny yellow plaques and firm-area thickening within the wall, the largest plaque measuring 0.4 cm.

**Figure 1:**
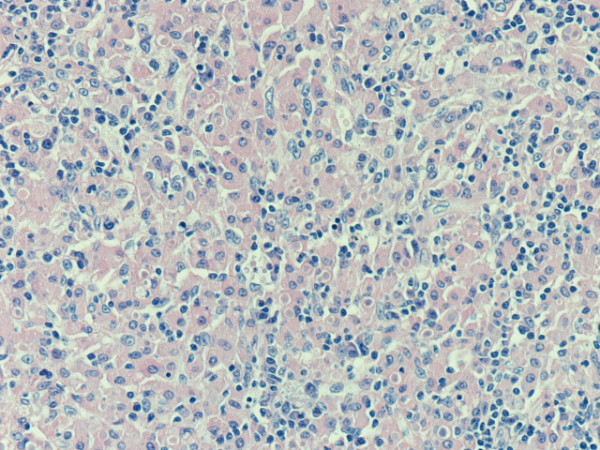
**Inflammatory cell infiltrate composed of histiocytes (von Hansemann cells)**. Haematoxylin and eosin, magnification ×20.

**Figure 2:**
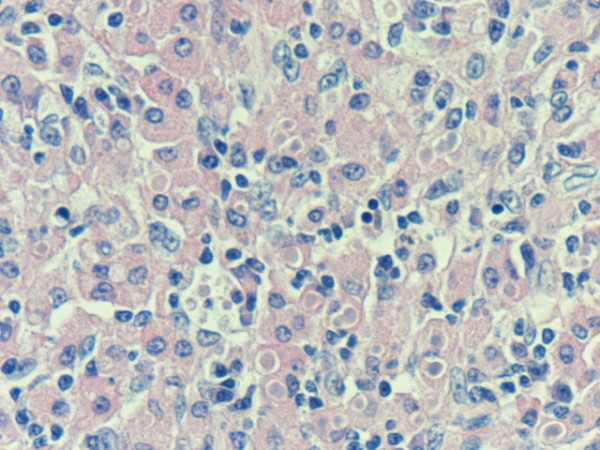
**Intracellular and extracellular Michaelis-Gutmann bodies surrounded by inflammatory cell infiltrate of histiocytes and lymphocytes**. Haematoxylin and eosin, magnification ×40.

On microscopy the colon showed typical ulcerative colitis with diffuse active inflammation, crypt abscesses and glandular architectural distortion. The appendix showed mucosal and submucosal dense collections of histiocytes with lymphocytes. The histiocytes (von Hansemann cells) had dense eosinophilic cytoplasm, vesicular nuclei and micronucleoli (Figure 1). These cells were positive with CD 68 and negative with S 100. There were round, basophilic intracellular inclusions (Michaelis-Gutmann bodies) of varying sizes, some calcified (Figure 2). Most of these inclusions were positive with the periodic acid-Schiff (PAS) stain after diastase digestion and focally with von Kossa and Perls' Prussian blue stains. Gram stain failed to reveal any bacteria.

## Discussion

The first human case of malakoplakia was described by von Hansemann, who coined the term 'malakoplakia' [1]. The aetiology of this condition is probably inflammatory. This benign non-neoplastic condition is believed to result from inadequate killing of bacteria, most commonly Escherichia coli, by a defect in monocytes and macrophages phagolysosomal activity. This response can also be seen with mycobacterial and fungal infections in immunocompromised patients. More than one organ can be affected simultaneously.

Clinically and macroscopically malakoplakia can simulate tumours or abscesses. Histologically sheets and aggregates of histiocytes (von Hansemann cells) with fine eosinophilic granular cytoplasm are seen on haematoxylin and eosin stain. They are characterised by intracellular and extracellular, round basophilic concretions, called the Michaelis-Gutmann (MG) bodies. These stain with PAS diastase, von Kossa stain and Perls' Prussian blue. Electron microscopically, MG bodies show concentric crystalline laminations with a dense central zone containing partially digested bacteria and a thin outer zone. Immunohistochemically the cells are positive with CD68, lysozyme and α-chymotrypsin.

Blackshear first reported Malakoplakia in the appendix in 1970 in a case of pulmonary nocardiosis [2]. Since then four more cases of malakoplakia of the appendix have been reported [3-5]. This includes an intriguing case of malakoplakia of the appendix associated with the eggs of Taenia species [5].

Although gastrointestinal malakoplakia is associated with a variety of conditions including ulcerative colitis, tuberculosis, diverticular disease, adenomas and carcinomas, these conditions have not been described with malakoplakia of the appendix. Histologically malakoplakia in the gastrointestinal tract (GIT) must be differentiated from Whipple's disease, other infectious and noninfectious granulomatous disorders and histiocyte storage diseases. The most common location for malakoplakia in the GIT is the colon and this is most commonly associated with carcinoma [6].

The exact aetiology of malakoplakia is ill understood. Malakoplakia is diagnosed primarily on histological grounds. Irrespective of the site, all malakoplakias share the same morphological features. Gram-negative bacteria, most commonly E. coli, have been frequently isolated from the cases of genitourinary malakoplakia. Various organisms that have been associated with this condition include E. coli, Mycobacterium tuberculosis, Shigella boydii, Paracoccidioides species, Rhodococcus equi, Yersinia enterocolitica, Klebsiella pneumoniae, Proteus mirabilis, Staphylococcus aureus, Pseudomonas aeruginosa, Enterobacter aerogenes and Taenia species [5]. The initial event in the pathogenesis of malakoplakia is partial digestion of the offending organism in macrophages by phagolysosomes. These are eventually damaged, resulting in calcification [7,8]. Some studies suggest that the underlying defect is a 3',5'-guanosine monophosphate dehydrogenase deficiency, causing diminished phagolysosomal and bactericidal activity.

Immunosuppressive conditions have been linked with malakoplakia. A case of ulcerative colitis treated with proctocolectomy showed immunoglobulins and muramidase within the malakoplakia histiocytes and an unusually high E. coli serum antibody titre [9].

Multiple blocks from the small and large intestine in our case failed to show malakoplakia that was just limited to the appendix and showed no other changes. The colon showed a reduced amount of mucosa associated lymphoid tissue (MALT) and extensive active inflammation and we propose that this may have led to the local immunosuppression, which allowed the organisms to be inefficiently cleared and proliferated. The patient's symptoms in our case were attributable to ulcerative colitis and not to malakoplakia, which was an incidental finding. No pre-operative clinical or radiological evidence of malakoplakia was found and the condition was diagnosed incidentally at histopathology. The underlying disease in our case was ulcerative colitis and the malakoplakia was limited to the appendix. The significance of this finding is not clear but we feel that this was a localised manifestation of the underlying immunosuppressive state. Ulcerative colitis and treatment with steroids may make a patient immunosuppressive and the local and systemic change in the immunity may facilitate the proliferation of the organisms and modify the phagocytic abilities of the macrophages.

## Conclusion

Malakoplakia of the appendix is extremely rare. Our case was associated with ulcerative colitis. Although the appendix showed malakoplakia, there were no features of ulcerative colitis within the appendix. We suggest the total volume of MALT was decreased, causing local immunosuppression in the appendix. This might have led to the local proliferation of organisms and defective phagocytic abilities of macrophages. Malakoplakia at all sites share the same histological features, with the presence of MG bodies being pathognomic. Malakoplakia can clinically simulate tumours and can be associated with tumours, infections and immunosuppression. Inefficient killing of the microorganism by the macrophages underpins the pathogenesis.

## Abbreviations

GIT: gastrointestinal tract; MALT: mucosa associated lymphoid tissue; MG: Michaelis-Gutmann; PAS: periodic acid-Schiff.

## Competing interests

The authors declare that they have no competing interests.

## Authors' contributions

SSS handled the specimen under the supervision of MCJS and reported the case and did a thorough literature search and designed this report, MCJS edited and revised the report critically and added required comments. All authors read and approved the final manuscript.

## Consent

Written informed consent was obtained from the patient for publication of this case report and any accompanying images. A copy of the written consent is available for review by the Editor-in-Chief of this journal
